# Determinants and regional inequalities in pregnancy deworming uptake in Somalia: findings from the 2020 Somalia demographic and health survey

**DOI:** 10.1016/j.ijregi.2026.100841

**Published:** 2026-01-06

**Authors:** Ahmed Saed Hussein, Ahmed Hassan Said, Mubarak Hassan Mohamud, Bashiir Abdirahman Guled, Sharif Abdi Mohamed, Ifrah Mukhtar Hussein

**Affiliations:** 1 Faculty of Medicine and Health Sciences, Jamhuriya University of Science and Technology, Mogadishu, Somalia; 2 Faculty of economics & management, Jamhuriya University of Science and Technology, Mogadishu, Somalia

**Keywords:** Deworming, Pregnancy, Somalia, Antenatal care, DHS, Soil-transmitted helminths

## Abstract

**Objectives:**

Soil-transmitted helminth (STH) infections remain widespread in Somalia, yet national evidence on deworming uptake during pregnancy is scarce. This study exams determinants and regional inequalities in pregnancy deworming uptake in Somalia: findings from the 2020 Somalia demographic and health survey (SDHS).

**Methods:**

This study analyzed data from 14,419 women in the 2020 SDHS. Weighted descriptive statistics, bivariate analyses, and multivariate logistic regression were applied to identify determinants of deworming drug use. All analyses accounted for survey clustering, stratification, and sampling weights.

**Results:**

National deworming coverage during pregnancy was 4.09%, among the lowest in sub-Saharan Africa. Uptake was significantly associated with antenatal care attendance (adjusted odds ratio [AOR] = 0.49), iron supplementation (AOR = 0.16), wealth (AOR = 1.81 among the richest), parity (AOR = 1.73), and maternal education (AOR = 1.72 for higher education). Marked geographic disparities were observed, with substantially higher odds in Lower Juba (AOR = 11.48), Bakool (AOR = 8.32), and Bay (AOR = 5.50) relative to Awdal. Nomadic women had increased odds of uptake (AOR = 6.01), while women in female-headed households were less likely to receive deworming (AOR = 0.86).

**Conclusions:**

Deworming uptake in Somalia is critically low and shaped by socioeconomic and regional inequities, underscoring the need to improve antenatal care access and drug supply.

## Highlights

• Deworming coverage during pregnancy in Somalia is extremely low at 4.09%.
• Antenatal care attendance, education, wealth, and iron supplementation are major predictors.
• Significant regional inequalities exceed tenfold differences in uptake.
• Nomadic women show higher odds of receiving deworming medication.
• Findings highlight urgent need to improve antenatal care access and drug supply systems.

## Introduction

Soil-transmitted helminth (STH) infections remain a persistent public health challenge across many low- and middle-income countries, disproportionately affecting populations living in poverty, displacement, and weak health-system environments [1]. Pregnant women are particularly vulnerable: chronic helminth infections contribute to maternal anemia, impaired nutritional status, and adverse birth outcomes, including low birthweight and neonatal mortality [2,3]. Although systematic reviews indicate that anthelminthic drugs, such as albendazole and mebendazole are safe after the first trimester and can improve maternal and neonatal outcomes [4,5], global coverage remains inconsistent. Recognizing these risks, the World Health Organization (WHO) recommends deworming after the first trimester as part of routine antenatal care (ANC) to support a positive pregnancy experience [6,7].

Despite these guidelines, the uptake of deworming during pregnancy across sub-Saharan Africa continues to be uneven and shaped by entrenched socioeconomic and structural inequities. Multi-country analyses show significant disparities by maternal education, wealth, ANC attendance, community context, and health-system access [8,9]. Country-specific studies echo these patterns: determinants consistently include ANC utilization, parity, media exposure, household decision-making dynamics, and geographic inequalities [[10], [11], [12]]. While these findings demonstrate the central role of ANC as a delivery point, they also highlight systemic failures especially in fragile contexts where weak service readiness, conflict, and displacement obstruct even basic maternal health interventions [13].

Somalia represents an extreme case of these structural constraints. Years of conflict, population displacement, protracted humanitarian crises, and chronic health workforce shortages have severely limited access to essential maternal health services [1]. Intestinal parasite exposure remains substantial, as documented among children in internally displaced persons (IDP) settlements in Mogadishu, where STH prevalence persists at levels that reinforce ongoing transmission [14]. Yet deworming coverage among pregnant women in Somalia has never been comprehensively examined at the national level. Only a recent subnational analysis from Somaliland provides preliminary evidence of socioeconomic and health-service factors influencing uptake [15], but its findings cannot be generalized to the wider Somali population due to differences in governance, health-system structure, and stability.

Globally, the picture is no more reassuring. Projections suggest that progress in deworming coverage among pregnant women remains far below what is required to meet 2030 targets, especially in conflict-affected settings [16]. The persistence of high STH burden, combined with the inadequacy of ANC coverage and inequities in the receipt of WHO-recommended interventions [[17], [18], [19]], underscores a systemic failure rather than a mere behavioral issue. Evidence from Ethiopia, Benin, Cameroon, and other African settings repeatedly demonstrates that individual behaviors are only one piece of the evidence chain—structural limitations, poverty, and geographic marginalization often exert a stronger influence on whether women receive deworming drugs [[20], [21], [22], [23]].

Somalia, situated in the Horn of Africa, faces particularly severe public health challenges due to decades of conflict, political instability, and recurrent humanitarian crises. These factors have severely compromised its healthcare infrastructure, limiting access to essential health services, including ANC and preventative interventions like deworming [24]. Recent research, such as studies in IDPs camps in Mogadishu, reveals a significant prevalence and intensity of intestinal and STH infections among children, indicating a broad community risk [14]. Additionally, a study focusing on Somaliland explored determinants of deworming during pregnancy, finding an alarmingly low overall prevalence of 1.9%, which is starkly below the WHO’s global coverage target of 75% [15].

Despite WHO recommendations and the documented health benefits of deworming during pregnancy, there remains a critical gap in research regarding the determinants influencing deworming drug use among pregnant women in Somalia. Existing studies are largely confined to small populations or specific regions like Somaliland, limiting their generalizability to the national context [15]. Furthermore, national-level data on crucial factors, such as maternal education, ANC attendance, wealth status, and regional disparities are scarce. This knowledge gap impedes the development of targeted interventions necessary to improve deworming coverage and maternal health outcomes. Therefore, this study aims to address this critical void by examining the determinants of deworming drug use during pregnancy using nationally representative data from the 2020 Somalia Demographic and Health Survey (SDHS), thereby providing vital insights for policymakers and public health programs. This study addresses that gap by analyzing the determinants and regional inequalities in pregnancy deworming uptake using data from the 2020 SDHS. By interrogating both individual and structural factors, the study provides evidence essential for policy direction in a context where preventable maternal morbidity remains unacceptably high and where deworming could serve as a low-cost, high-impact intervention—if inequitable access barriers are understood and addressed.

## Methods

### Study design and data source

This study employed a cross-sectional analytical design using data from the 2020 SDHS. The SDHS is a nationally representative survey implemented by the Federal Government of Somalia with technical support from the DHS Program. It uses standardized instruments across countries, allowing comparability with regional and global evidence. The survey collects detailed information on maternal health behaviors, child health indicators, fertility, household characteristics, and service utilization.

To focus specifically on pregnancy-related interventions, the analysis was restricted to women aged 15-49 who had a live birth in the 5 years preceding the survey. Only the most recent pregnancy was considered to minimize recall bias and ensure consistency with DHS analytical practice.

### Sampling strategy

The SDHS utilized a two-stage stratified cluster sampling design. In the first stage, enumeration areas were selected using probability proportional to size. In the second stage, households were systematically sampled within each selected cluster. Sampling was stratified across urban, rural, and nomadic populations, and Somalia’s major administrative regions.

To generate unbiased population estimates, all analyses incorporated sampling weights, primary sampling units (PSUs), and stratification variables following DHS analytical guidelines.

### Study variables

#### Outcome variable

The dependent variable was use of deworming medication during pregnancy, coded as a binary indicator (1 = received deworming drug; 0 = did not). These variable captures whether the respondent reported taking albendazole, mebendazole, or any other anthelminthic drug during ANC for her most recent pregnancy.

#### Independent variables

Variables were selected based on theoretical relevance, prior empirical evidence, and WHO recommendations on maternal health determinants. These included: **Socio-demographic factors**: maternal age group, education level, parity, household wealth index, sex of household head, respondent employment. **Contextual and structural factors**: region, type of residence (urban, rural, nomadic), perceived distance to health facility (“big problem” vs “not a big problem”). **Maternal health service factors**: ANC attendance (at least 1 visit vs none), iron supplementation, pregnancy intention. **Media exposure**: frequency of radio and television use.

All independent variables correspond to the recoded categories presented in the SDHS dataset (see weighted sample distribution and logistic regression results).

### Data analysis

Analysis was conducted in three stages:

#### Descriptive analysis

Weighted frequencies and percentages were calculated to describe the sample distribution across socio-demographic, structural, and service-related characteristics. This step identified baseline disparities in the uptake of deworming medication.

#### Bivariate analysis

Crude associations between each independent variable and the outcome were examined using **binary logistic regression**. Variables with *P* <0.20 in bivariate analysis were retained for multivariate modeling to avoid premature exclusion of potentially relevant predictors.

#### Multivariate logistic regression

A multivariate logistic regression model was fitted to identify independent determinants of deworming uptake. Adjusted odds ratios (AORs) with 95% confidence intervals (CIs) were reported. The model accounted for survey design using the DHS “svy” estimation procedures.

The final model included socioeconomic, demographic, service-related, and regional predictors, consistent with DHS and global deworming literature. Findings revealed substantial regional and contextual inequalities, including strong effects for nomadic residence, wealth, parity, ANC attendance, and iron use.

### Ethical considerations

The SDHS obtained ethical clearance from national authorities and the ICF International Institutional Review Board. This study involved secondary analysis of anonymized data and required no additional ethical approval. No personally identifying information was accessed at any stage.

### Methodological rigor and assumptions

Several methodological decisions were made to preserve analytical integrity:

• Survey weighting and clustering adjustments were strictly applied to avoid biased estimates.
• Only the most recent birth was analyzed to minimize recall error.
• Multicollinearity among predictors was assessed; no variance inflation factor exceeded acceptable thresholds.
• All significance tests used a two-sided α = 0.05.

## Results

### Prevalence of deworming

Only 4.09% of pregnant women received deworming medication, while 95.91% did not. This represents one of the lowest national coverage levels reported in sub-Saharan Africa.

### Sample characteristics

A total weighted sample of 14,419 women with a recent live birth was analyzed. The majority resided in urban areas (62.1%), while 25.3% lived in rural settings and 12.6% in nomadic communities. Educational attainment was very low, with 85.0% reporting no formal education. Nearly half of households (47.3%) were in the poorest wealth quintile, and 88.4% of women were multiparous.

Access to maternal services was limited: 68.9% reported no ANC visit during pregnancy, 73.2% did not take iron supplements, and over 91% had no media exposure through radio or television.

Deworming during pregnancy was extremely rare: 4.09% reported receiving deworming medication (95.91% did not).

### Multivariable determinants

Nomadic women had significantly higher odds of receiving deworming medication (AOR = 6.01; 95% CI: 4.86-7.44). This result is counterintuitive, given the mobility barriers often associated with nomadic populations. However, this could be explained by targeted health outreach programs designed for mobile communities, such as mobile health clinics and community health workers, which help provide services in areas with difficult access.

In contrast, urban women were less likely to receive deworming compared to rural women (AOR = 0.77; 95% CI: 0.67-0.89). This suggests that urban health services may be facing issues like inequitable distribution of resources or socioeconomic disparities, which may limit access to preventive services like deworming, even though urban areas typically have better healthcare infrastructure.

Women with formal schooling were significantly more likely to be dewormed, with the effect strengthening at higher levels of education: Secondary education (AOR = 1.41; 95% CI: 1.11-1.78; *P* = 0.006), Higher education (AOR = 1.72; 95% CI: 1.23-2.40; *P* = 0.002). This highlights the strong association between education and health literacy, which drives health-seeking behaviors and increases the likelihood of using preventive services like deworming.

Interestingly, primary education did not maintain significance after adjustment (*P* = 0.322), suggesting that only higher levels of education confer a meaningful advantage in terms of deworming uptake, possibly due to increased awareness of health risks and access to services.

Wealth was a strong and graded predictor of deworming uptake. Compared to the poorest women: Middle-income women had 43% higher odds (AOR = 1.43; *P* <0.001). Women in the highest wealth quintile had 81% higher odds (AOR = 1.81; *P* <0.001).

This socioeconomic gradient demonstrates that deworming access is deeply unequal.

Multiparous women had significantly higher odds of receiving deworming medication (AOR = 1.73; 95% CI: 1.48-2.02; *P* <0.001). This pattern suggests increasing familiarity with ANC services or prior positive experiences influencing subsequent pregnancies.

Iron use was the single strongest predictor of deworming uptake. Women who did not use iron supplements were 84% less likely to receive deworming treatment (AOR = 0.16; 95% CI: 0.12-0.22; *P* <0.001).

This strong correlation indicates that deworming is functionally tied to ANC service delivery quality. Women who did not attend ANC had markedly reduced odds of receiving deworming medication: AOR = 0.49 (95% CI: 0.38-0.62; *P* <0.001). This finding suggests that ANC attendance serves as a crucial gateway to deworming, supporting the idea that ANC is the primary and likely only point of deworming delivery in Somalia. Despite these findings, the low levels of ANC attendance (68.9% of the sample did not attend ANC) likely reflect service delivery gaps, such as limited drug availability, inadequate outreach, or inconsistent service provision, which may have led to the observed disparities in deworming uptake.

Unemployed women were significantly less likely to receive deworming medication

(AOR = 0.64; 95% CI: 0.49-0.83; *P* = 0.001), reflecting economic barriers and possibly reduced autonomy.

Women living in female-headed households had lower odds of deworming uptake (AOR = 0.86; 95% CI: 0.79-0.92; *P* <0.001). This suggests structural vulnerabilities beyond simple economic disadvantage.

Unexpectedly, women with *unwanted pregnancies* had higher odds of receiving deworming medication (AOR = 1.56; 95% CI: 1.16-2.10; *P* = 0.004). This contradicts traditional assumptions and indicates a need for closer examination of health-seeking dynamics.

### Regional inequalities

The regional disparities in deworming uptake observed in this study are significant and require a more nuanced explanation. For instance, Lower Juba (AOR = 11.48) and Bakool (AOR = 8.32) exhibit the highest odds of deworming uptake compared to Awdal, reflecting the role of targeted NGO programs and humanitarian interventions that may be more prominent in conflict-affected areas. In these regions, mobile health clinics, community outreach efforts, and better health system support from NGOs likely contribute to the higher uptake.

Moreover, regional variations in health infrastructure and conflict intensity also play critical roles. Regions like Lower Juba and Bay, with higher odds, have been prioritized for humanitarian aid due to displacement and ongoing conflict, which may have led to better access to maternal health services, including deworming. In contrast, regions with less health system capacity or those facing greater geopolitical instability, such as Awdal, may experience lower deworming uptake due to limited outreach or supply chain challenges. These findings underscore the need for region-specific interventions that consider local health service readiness, conflict-related factors, and the role of NGOs in overcoming barriers to deworming services.

Several regions (e.g., Woqooyi Galbeed, Sool, Galgaduud) did not retain significance, highlighting heterogeneity in service delivery and outreach. These disparities reflect structural rather than behavioral determinants likely tied to localized health programs, NGO presence, conflict intensity, and mobility patterns.

## Discussion

This study examined the prevalence and determinants of deworming drug use during pregnancy in Somalia using data from the 2020 SDHS. Deworming coverage was 4.09%, which is extremely low compared with other countries in sub-Saharan Africa. Similarly low levels have been reported in settings, such as Angola (2.0%), Zimbabwe (3.6%), and Ethiopia (5.7%) [8]. However, substantially higher coverage has been documented elsewhere, including 16.5% among Ethiopian women with ANC visits [21], 29.8% in Cameroon [8], and regional estimates of 67.6% in East Africa, 50.7% in Central Africa, and 24.3% in West Africa [25]. Country-level estimates, such as 84.1% in Sierra Leone, 77.8% in Zambia, and 71.6% in Gabon further illustrate the contrast. Across 49 STH-endemic countries, the average coverage is 23% [21]. These comparisons underscore that Somalia’s uptake remains among the lowest globally. ANC attendance was one of the strongest predictors of deworming uptake. Women who attended at least one ANC visit were significantly more likely to receive deworming medication than those who did not. This finding is consistent with evidence from Ethiopia, Kenya, and Uganda, where ANC serves as the primary platform for delivering maternal preventive interventions, such as deworming, iron supplementation, and tetanus immunization [9,16]. Given that deworming tablets are normally distributed through ANC, limited contact with ANC services in Somalia likely contributes to low national coverage.

Education was also positively associated with deworming uptake. Women with secondary or higher education had significantly greater odds of receiving deworming medication compared with those with no formal schooling. Comparable associations have been documented in Ghana and Uganda, where education increases health literacy, care-seeking behavior, and engagement with maternal health services [16,21]. In Somalia, where women’s educational attainment remains low, targeted communication strategies may be needed to reach women with limited formal schooling.

Household wealth showed a clear gradient, with women from middle- and high-income households having higher odds of deworming uptake than those from poorer households. This aligns with findings from Nigeria, Ethiopia, and Tanzania suggesting that economic disparities shape access to ANC and essential medicines [18,24]. Evidence from Somalia indicates persistent income-related inequalities in health service utilization and nutrition outcomes [14,26], which likely influence access to ANC-based interventions, such as deworming. Parity was another significant factor: multiparous women were more likely to receive deworming medication than primiparous women, reflecting patterns observed in Kenya and Ghana [9,16]. This association may indicate that previous experiences with pregnancy and the health system facilitate subsequent service use.

Significant regional disparities were also observed. Women in Hiraan, Banadir, Bay, Bakool, Gedo, and Lower Juba had substantially higher odds of receiving deworming medication compared with those in Awdal. Similar regional differences have been documented in other East African contexts where health service coverage and infrastructure vary widely [21,24]. In Somalia, these patterns may reflect differences in health system capacity, program implementation, and support from development partners [1,15]. Overall, the findings show that Somalia’s extremely low deworming coverage is shaped by socioeconomic, service-access, and regional inequities rather than individual-level factors alone.

### Strengths

Use of a large, nationally representative dataset (SDHS 2020) enhances generalizability.

Application of survey-adjusted multivariate regression allows robust estimation of determinants.

First study to examine nationwide regional inequalities in maternal deworming coverage in Somalia.

### Limitations

Cross-sectional design limits causal interpretation.

Self-reported deworming and ANC variables may be affected by recall bias.

DHS does not capture data on facility readiness, supply stockouts, or NGO program presence—factors likely influencing regional disparities.

Timing of deworming (by trimester) is not documented.

Nomadic populations may be underrepresented due to mobility challenges in data collection.

## Conclusion

This study provides the first national-level evidence on the determinants of deworming drug use during pregnancy in Somalia. The findings reveal that uptake remains extremely low at **4.09%**, far below regional and global averages. Deworming access is determined not by individual preference but by structural constraints limited ANC contact, low education, economic vulnerability, and profound regional disparities. The strongest predictors (ANC attendance and iron supplementation) demonstrate that deworming is embedded within ANC service readiness. Regions with better health system support exhibit far higher uptake, while underserved areas remain left behind.

Improving deworming coverage in Somalia will require systemic efforts: expanding ANC availability, addressing socioeconomic barriers, strengthening supply chains, and ensuring equitable regional implementation. Without targeted investments, Somalia will continue to fall short of WHO recommendations and miss an opportunity to reduce preventable maternal morbidity linked to helminth infections (Tables 1 and 2).

**Table 1:** Sample characteristics.

Variable	Categories	Weighted frequencies	Percent
Total sample		14,419.20	100
Age group of respondents	15-24	3833.22	26.58
	25-34	6953.57	48.22
	35-49	3632.40	25.19
Region	Awdal	304.81	2.11
	Woqooyi Galbeed	1349.28	9.36
	Togdheer	795.54	5.52
	Sool	457.1	3.17
	Sanaag	568.66	3.94
	Bari	921.43	6.39
	Nugaal	438.25	3.04
	Mudug	985.37	6.83
	Galgaduud	900.92	6.25
	Hiraan	542.83	3.76
	Middle Shabelle	1016.65	7.05
	Banadir	3598.39	24.96
	Bay	739.52	5.13
	Bakool	361.73	2.51
	Gedo	641.39	4.45
	Lower Juba	797.34	5.53
Type of place of residence	Rural	3650.88	25.32
	Urban	8956.39	62.11
	Nomadic	1811.92	12.57
Highest educational level	No Education	12,259.55	85.02
	Primary	1644.19	11.4
	Secondary	380.26	2.64
	Higher	135.19	0.94
Sex of household head	Male	9901.24	68.67
	Female	4517.95	31.33
Frequency of listening to radio	Yes	1166.05	8.09
	No	13,253.15	91.91
Frequency of watching television	Yes	1261.79	8.75
	No	13,157.41	91.25
Wealth quintile	Low	6823.64	47.32
	Middle	2605.73	18.07
	High	4989.82	34.61
Parity category	Primiparous	1666.60	11.56
	Multiparous	12,752.59	88.44
Distance to health facility: Getting medical help for self	Big Problem	9396.36	65.17
	Not big problem	5022.84	34.83
Husband/partner worked in last 12 months	Yes	5809.10	40.29
	No	8610.09	59.71
Pregnancy desire	Wanted	12,993.68	90.11
	Unwanted	1425.52	9.89
Attended ANC at least once	Had ANC visit	4484.52	31.1
	No ANC visit	9934.68	68.9
Iron use during pregnancy	Yes	3869.48	26.84
	No	10,549.71	73.16

ANC, antenatal care.

**Table 2:** Logistic regression outputs.

Variable	Category (Ref)	Crude OR (95% CI)	*P*-value	Adjusted OR (95% CI)	*P*-value
Residence	Rural (Ref)				
	Urban	1.02 (0.75-1.41)	0.879	0.77 (0.67-0.89)	<0.001
	Nomadic	3.62 (2.61-5.02)	<0.001	6.01 (4.86-7.44)	<0.001
Education	No education (Ref)				
	Primary	1.50 (1.12-1.99)	0.006	0.84 (0.60-1.19)	0.322
	Secondary	3.17 (2.48-4.05)	<0.001	1.41 (1.11-1.78)	0.006
	Higher	3.19 (2.19-4.64)	<0.001	1.72 (1.23-2.40)	0.002
Wealth Index	Low (Ref)				
	Middle	3.39 (2.75-4.18)	<0.001	1.43 (1.28-1.60)	<0.001
	High	3.93 (3.19-4.84)	<0.001	1.81 (1.59-2.06)	<0.001
Parity	Primiparous (Ref)				
	Multiparous	1.59 (1.34-1.89)	<0.001	1.73 (1.48-2.02)	<0.001
Iron Use	Yes (Ref)				
	No	0.10 (0.09-0.12)	<0.001	0.16 (0.12-0.22)	<0.001
ANC Visit	Yes (Ref)				
	No	0.20 (0.18-0.22)	<0.001	0.49 (0.38-0.62)	<0.001
Respondent Employment	Yes (Ref)				
	No	0.37 (0.26-0.54)	<0.001	0.64 (0.49-0.83)	0.001
Household Head Sex	Male (Ref)				
	Female	0.76 (0.70-0.84)	<0.001	0.86 (0.79-0.92)	<0.001
Pregnancy Desire	Wanted (Ref)				
	Unwanted	0.93 (0.69-1.26)	0.641	1.56 (1.16-2.10)	0.004
Region	Awdal (Ref)				
	Woqooyi Galbeed	0.25 (0.06-1.05)	0.058	0.52 (0.24-1.12)	0.095
	Togdheer	1.20 (0.41-3.48)	0.737	1.01 (0.57-1.77)	0.978
	Sool	1.32 (0.51-3.43)	0.56	0.61 (0.33-1.09)	0.096
	Sanaag	2.71 (0.95-7.71)	0.062	0.99 (0.57-1.72)	0.981
	Bari	0.52 (0.14-1.89)	0.319	1.08 (0.51-2.29)	0.831
	Nugaal	0.70 (0.24-2.05)	0.515	1.52 (0.87-2.64)	0.139
	Mudug	0.68 (0.25-1.85)	0.447	1.22 (0.68-2.17)	0.502
	Galgaduud	0.28 (0.08-0.95)	0.041	0.56 (0.29-1.09)	0.09
	Hiraan	0.87 (0.34-2.18)	0.757	2.12 (1.19-3.78)	0.011
	Middle Shabelle	0.68 (0.27-1.69)	0.399	1.35 (0.79-2.31)	0.265
	Banadir	0.86 (0.35-2.11)	0.748	2.93 (1.70-5.05)	<0.001
	Bay	2.28 (0.94-5.55)	0.07	5.50 (3.20-9.44)	<0.001
	Bakool	3.94 (1.54-10.07)	0.005	8.32 (4.44-15.59)	<0.001
	Gedo	0.96 (0.39-2.33)	0.923	4.96 (2.87-8.57)	<0.001
	Lower Juba	3.49 (1.43-8.51)	0.006	11.48 (6.57-20.07)	<0.001

Note: Adjusted ORs in bold indicate statistical significance (*P* <0.05).

CI, confidence interval; OR, odds ratio; Ref, reference category.

## Policy implications for improving deworming uptake in Somalia

1) Strengthening ANC service delivery:
  • Action: Prioritize improving ANC access in both urban and rural areas through the provision of mobile health units and outreach programs.
  • Context: Many women in Somalia, particularly in rural or conflict-affected regions, struggle to access ANC services. Mobile clinics and community health workers should be deployed to reach underserved areas, particularly where health facilities are distant or inadequately staffed.
  • Example: Mobile ANC clinics could be expanded to reach remote communities to provide deworming medication along with routine maternal health services.
2) Targeted outreach programs for nomadic populations:
  • Action: Develop nomadic health programs that ensure continuous access to health services, including deworming and nutritional support. These should include mobile health units and community-based health interventions tailored for nomadic lifestyles.
  • Context: Nomadic women, while often overlooked in public health interventions, may benefit from customized outreach programs that respect their lifestyle and mobility.
  • Example: NGOs or international health agencies could collaborate to provide seasonal mobile health teams that offer deworming medication, nutritional support, and basic health services for nomadic communities.
3) Integrating health services with nutrition programs:
  • Action: Integrate deworming programs with nutrition initiatives, particularly in IDP camps and regions with high malnutrition rates. Collaboration between nutrition programs and maternal health services can help address the underlying causes of poor health and improve overall health outcomes.
  • Context: Given the high malnutrition rates in Somalia, integrating deworming and nutrition services could enhance the effectiveness of both.
  • Example: Partner with UNICEF, WFP, and local NGOs to provide nutritional interventions and deworming services simultaneously, particularly for pregnant women and children in IDP camps and marginalized communities.
4) Improving health education and awareness:
  • Action: Launch national and community-based health education campaigns that raise awareness of the benefits of deworming and encourage health-seeking behaviors. Focus on increasing awareness among women with low education levels and communities with limited access to healthcare services.
  • Context: Education plays a critical role in improving health outcomes. By targeting low-literacy populations, you can increase deworming coverage and improve maternal and child health.
  • Example: Develop radio programs, local workshops, and community health sessions that educate people about the importance of deworming and preventive care, using culturally appropriate messages.
5) Enhancing the role of humanitarian actors:
  • Action: Strengthen the coordination between local governments, NGOs, and international organizations to improve health service delivery in conflict zones and IDP camps. Establish clear protocols for providing deworming as part of emergency health responses.
  • Context: Somalia’s ongoing conflict and the large number of displaced people complicate health service delivery. Humanitarian actors must play a key role in reaching vulnerable populations in areas with limited government presence.
  • Example: Humanitarian agencies like MSF and UNICEF could work with the Somali Ministry of Health to set up temporary health facilities in IDP camps that provide deworming along with basic maternal health services.

## Declaration of competing interest

The author have no competing interests to declare.

## References

[1] World Health Organization (2023). Somalia: Health Cluster Bulletin December 2023. https://reliefweb.int/report/somalia/somalia-health-cluster-bulletin-december-2023.

[2] Lau R, Chris RB, Phuong MS, Khatib A, Kopalakrishnan S, Bhasker S, et al. Treatment of soil-transmitted helminth infections in pregnancy: a systematic review and meta-analysis of maternal outcomes. 2020;27:taz079. 10.1093/jtm/taz079/5602451. 31641774

[3] Walia B., Kmush B.L., Lane S.D., Endy T., Montresor A., Larsen DA. (2021). Routine deworming during antenatal care decreases risk of neonatal mortality and low birthweight: a retrospective cohort of survey data. PLoS Negl Trop Dis.

[4] Gyorkos T.W., St-Denis K. (2019). Systematic review of exposure to albendazole or mebendazole during pregnancy and effects on maternal and child outcomes, with particular reference to exposure in the first trimester. Int J Parasitol.

[5] Salam R.A., Cousens S., Welch V., Gaffey M., Middleton P., Makrides M. (2019). Mass deworming for soil-transmitted helminths and schistosomiasis among pregnant women: a systematic review and individual participant data meta-analysis. Campbell Syst Rev.

[6] World Health Organization (2016). WHO recommendations on antenatal care for a positive pregnancy experience. https://www.who.int/publications/i/item/9789241549912.

[7] World Health Organization (2023). Deworming in pregnant women. https://www.who.int/tools/elena/interventions/deworming-pregnancy?utm_source=chatgpt.com.

[8] Zegeye B., Keetile M., Ahinkorah B.O., Ameyaw E.K., Seidu A.A., Yaya S. (2021). Utilization of deworming medication and its associated factors among pregnant married women in 26 sub-Saharan African countries: a multi-country analysis. Trop Med Health.

[9] Aragaw F.M., Belay D.G., Endalew M., Asratie M.H., Gashaw M., Tsega NT. (2022). Individual and community level predictors of utilization of deworming medications among pregnant women in Ethiopia: a multilevel analysis. PLoS Negl Trop Dis.

[10] Amoak D., Dhillon S., Antabe R., Sano Y., Luginaah I. (2023). Factors associated with deworming medication utilization among pregnant women in Benin: evidence from the demographic and health survey. Trop Med Infect Dis.

[11] Bankanie V., Moshi FV. (2022). Factors associated with the use of deworming drugs during pregnancy in Tanzania; an analysis from the 2015–16 Tanzanian HIV and malaria indicators survey. BMC Pregnancy Childbirth.

[12] Osborne A., Bai-Sesay A.U., Tommy A., Bangura C., Ahinkorah B.O. (2024). Trends and inequalities in the use of deworming medication during pregnancy in Sierra Leone, 2008–2019. Trop Med Health.

[13] Tancred T., Mubangizi V., Dei E.N., Natukunda S., Abankwah D.N., Ellis P. (2024). Prevention and management of anaemia in pregnancy: community perceptions and facility readiness in Ghana and Uganda. PLOS Global Public Health.

[14] Garba B., Asowe H.A., Dirie N.I., Umar Y., Salah A.O., Hussien A.A. (2025). Prevalence and intensity of intestinal and soil-transmitted helminths infection among children in internally displaced camps in Mogadishu Somalia. Sci Rep.

[15] Mohamed A.A., Ali S.A., Yasin A.I., Mahdi A.H., Ahmed A.M., Aden A.A. (2025). Determinants of deworming during pregnancy in somaliland: a binary and multivariate logistic regression analysis. Discover Social Science Health.

[16] Sassa M., Yoneoka D., Ng C.F., Cao A.Q., Devanathan G., Hashizume M. (2024). A comprehensive assessment of deworming coverage among pregnant women in low- and middle-income countries, 2000–30. J Glob Health.

[17] Ayele B.A., Holliday E., Chojenta C. (2025). Determinants of antenatal care service utilisation in sub-Saharan Africa: an analysis of demographic and health surveys data (2015–2022). Arch Public Health.

[18] Mebratie AD. (2024). Receipt of core antenatal care components and associated factors in Ethiopia: a multilevel analysis. Front Glob Womens Health.

[19] Gebeyehu A.A., Dessie A.M., Zemene M.A., Anteneh R.M., Chanie E.S., Kebede N. (2024). Inadequacy of antenatal care attendance and its determinants amongst pregnant women in Ethiopia based on the 2019 Mini-Ethiopian demographic health survey: secondary data analysis. BMC Pregnancy Childbirth.

[20] Admasie C., Wasie B., Abeje G. (2014). Determinants of prescribed drug use among pregnant women in Bahir Dar city administration, Northwest Ethiopia: a cross sectional study. BMC Pregnancy Childbirth.

[21] Bantie B., Kassaw Yirga G., Ayenew Y.E., Nuru Muhamed A., Tassew S.F., Kassie Y.T. (2023). Deworming utilization among pregnant mothers with at least one antenatal care follow-up in Ethiopia, 2022:- A multilevel analysis. PLoS One.

[22] Mulaw G.F., Feleke F.W., Ahmed S.S., Bamud JA. (2021). Deworming coverage and its predictors among Ethiopian children aged 24 to 59 months: further analysis of EDHS 2016 data set. Glob Pediatr Health.

[23] Terefe B., Jembere M.M., Assimamaw N.T., Chekole B. (2024). Deworming coverage and its determinants among 12-59 months old children in East Africa: a population-based study. PLoS One.

[24] World Health Organization (2023). Soil-transmitted helminth infections. https://www.who.int/news-room/fact-sheets/detail/soil-transmitted-helminth-infections?utm_source=chatgpt.com.

[25] Zegeye B., Ahinkorah B.O., Ameyaw E.K., Seidu A.A., Yaya S. (2021). Utilization of deworming drugs and its individual and community level predictors among pregnant married women in Cameroon: a multilevel modeling. Biomed Res Int.

[26] UNICEF (2024).

